# Coronary perfusion pressure in a pig model of prolonged cardiac arrest treated by different modes of venoarterial extracorporeal membrane oxygenation and intraaortic balloon counterpulsation

**DOI:** 10.1186/cc10880

**Published:** 2012-03-20

**Authors:** J Bělohlávek, M Mlcek, M Huptych, S Havranek, P Ostadal, A Linhart, O Kittnar

**Affiliations:** 1 General Teaching Hospital Prague, Czech Republic; 2 1st Medical School, Charles University, Prague, Czech Republic; 3 Technical Institute Prague, Czech Republic; 4 Homolka Hospital, Prague, Czech Republic

## Introduction

An extracorporeal membrane oxygenation (ECMO)-based approach is increasingly used in cardiac arrest (CA). However, little is known about coronary perfusion pressure progress over time in CA managed by ECMO. The aim of this study was to assess femorofemoral (FF) compared to femoro-subclavian (FS) venoarterial ECMO in a pig model of prolonged CA on coronary perfusion pressure (CPP), myocardial metabolic recovery and resuscitability.

## Methods

A total of 11 female pigs, body weights 50.3 ± 3.4 kg, were enrolled into a protocol of prolonged cardiac arrest treated by FF or FS ECMO ± IABP in a randomized fashion. Animals under general anesthesia had undergone 15 minutes of ventricle fibrillation (VF) with basal ECMO flow of 5 to 10 ml/kg/minute simulating low-flow CA followed by continued VF with ECMO flow of 100 ml/kg/minute. CPP, myocardial lactate metabolism and myocardial oxygen extraction were determined.

## Results

CPP decreased from baseline of 85 mmHg (72, 94.5) to 15 mmHg (10, 20.5) during CA. The first CPP value on ECMO increased to 34 mmHg (26.5, 44) and during the further protocol gradually rose to significantly higher CPP of 68 mmHg (45.5, 82) before CPR (*P *= 0.003). This phenomenon of gradual rise was even more pronounced in FF ECMO, animals started on FF ECMO completed the protocol with identical CPP values as at baseline (85 mmHg (80, 99) vs. 86 mmHg (78, 86), *P *= 0.55). Following CA, significantly higher lactate levels were detected in animals started on FS ECMO in all post-arrest periods (*P *= 0.016 and *P *= 0.035 for difference in arterial and coronary sinus lactate levels, respectively). Oxygen extractions after a steep increase during CA declined immediately after ECMO initiation and remained further with no statistically significant differences between respective ECMO arms (*P *for difference = 0.547). Resuscitability was high, we gained 5 minutes return of spontaneous circulation (ROSC) in eight animals (73%) and 60 minutes ROSC was present still in eight animals (73%).

## Conclusion

Our experimental study confirmed that, in a pig model of prolonged cardiac arrest, VA ECMO, mainly the FF approach, increases significantly the CPP over time, assures good metabolic recovery and offers sustained reasonable resuscitability.

